# Pregnancy Rate Is High When the Length of the Luteal Phase During the In Vitro Fertilization Hormone Replacement Cycle Is 144 Hours or More Before Embryo Transfer

**DOI:** 10.7759/cureus.81185

**Published:** 2025-03-25

**Authors:** Eiji Nishio, Shota Oikawa, Eriko Sakakibara, Miho Ishikawa, Kiriko Kotani, Hikari Yoshizawa, Hironori Miyamura, Takanori Hayashi, Haruki Nishizawa

**Affiliations:** 1 Obstetrics and Gynecology, Fujita Health University, Toyoake, JPN; 2 Clinical Laboratory, Fujita Health University, Toyoake, JPN; 3 Anatomy and Medical Biology, Fujita Health University, Toyoake, JPN

**Keywords:** assisted reproductive technology, dydrogesterone, endometrial receptivity, frozen-thawed embryo transfer, hormone replacement cycle, pregnancy rate, window of implantation

## Abstract

Background: When using assisted reproductive technology, there are cases where, despite the transfer of a good embryo, sometimes pregnancy may not be the case. Thus, during hormone replacement cycle implantation, it is important to synchronize the number of days of progesterone administration with the degree of embryo maturity. This study aimed to compare the outcomes of the administration of oral dydrogesterone for the duration of progestin use during the hormone replacement cycle for frozen-thawed blastocyst transfer.

Material and methods: The primary outcome of this study was the clinical pregnancy rate. We performed a retrospective cohort study of patients who underwent frozen-thawed blastocyst transfers between January 2017 and December 2024. According to our standard protocol, a vitrified-warmed blastocyst transfer was performed using dydrogesterone, which was administered orally at our center. A total of 554 cases were included in the study. Using the Gardner classification to evaluate the quality of blastocysts, grade AA was classified as the best quality, the AB/BA group as good quality, and the BB group as fair quality. We classified the 554 cases into 317 AA, 163 AB/BA, and 74 BB cases using the Gardner classification. Based on the duration of progestin administration, patients were divided into four groups: 120 hours (120 h), 132 hours (132 h), 144 hours (144 h), and 156 hours (156 h). We used the Shapiro-Wilk method and the Steel-Dwass test to determine whether there were differences in patients' background age and BMI among the four groups (120 h, 132 h, 144 h, and 156 h). We used Fisher's exact test and the Bonferroni method to determine whether there were differences in the final outcome of pregnancy rate between the four groups of 120 h, 132 h, 144 h, and 156 h.

Results: In the analysis of all embryos, the pregnancy rate at each timepoint of the primary evaluation was significantly higher in the 144-h group than in the 132-h group. Next, on analyzing the results by embryo grade, there was no difference in the pregnancy rate at each timepoint in the AA group. In the AB/BA group, the pregnancy rate was higher in the 144-h group than in the 132-h group. In the BB group, the pregnancy rate was higher in the 144-h group than in the 132-h group.

Conclusion: This study clarified two aspects. First, the pregnancy rate in the 144-h group was significantly higher than that in the 132-h group in the analysis of all embryos. Second, the window of implantation may be more important for poor-quality embryos. This study showed that the oral administration of dydrogesterone requires a window of implantation of at least 144 hours.

## Introduction

When using assisted reproductive technology, there are cases in spite of the transfer of a good embryo, sometimes pregnancy may not be the case. In recent years, preimplantation genetic diagnosis has been introduced, and endometrial factors have attracted attention as a cause of pregnancy failure, even when euploid embryos are transferred. The clinical pregnancy rate per embryo transfer in which a preimplantation genetic diagnosis was performed was reported to be 54.4% [1], suggesting that factors other than embryo quality are necessary for a successful pregnancy.

During hormone replacement cycle implantation, it is important to synchronize the number of days of progesterone administration with the degree of embryo maturity. The clinical translation of endometrial transcriptomics has provided an objective definition of the limited period during which the maternal endometrium is receptive to an embryo, known as the window of implantation (WOI) [2]. Although there is little clear supportive evidence, the duration of progestin use during a hormone replacement cycle is often 5-7 days.

Dydrogesterone has long been used to treat conditions associated with progesterone deficiency. Dydrogesterone is a 6-dihydro-retroprogesterone characterized by high oral bioavailability, good tolerability, easy absorption, and high selectivity for progesterone receptors [3]. It was developed in the 1960s and has been shown to be effective in relieving dysmenorrhea [4-6]. To date, only a few studies have examined the use of oral dydrogesterone for luteal phase support in fresh in vitro fertilization cycles, with variable results summarized in a recent review [7]. 

In recent years, two randomized controlled clinical trials have demonstrated that oral dydrogesterone, an artificial progesterone derivative, is non-inferior in terms of pregnancy rate at 12 weeks of gestation and could be an alternative option [8,9]. However, to the best of our knowledge, there are no reports on the most favorable duration of dydrogesterone administration during the in vitro fertilization hormone replacement cycle before embryo transfer. Therefore, this study aimed to compare the outcomes of an administration protocol of oral dydrogesterone for the duration of progestin use during a hormone replacement cycle for frozen-thawed blastocyst transfer.

## Materials and methods

**Figure 1:**
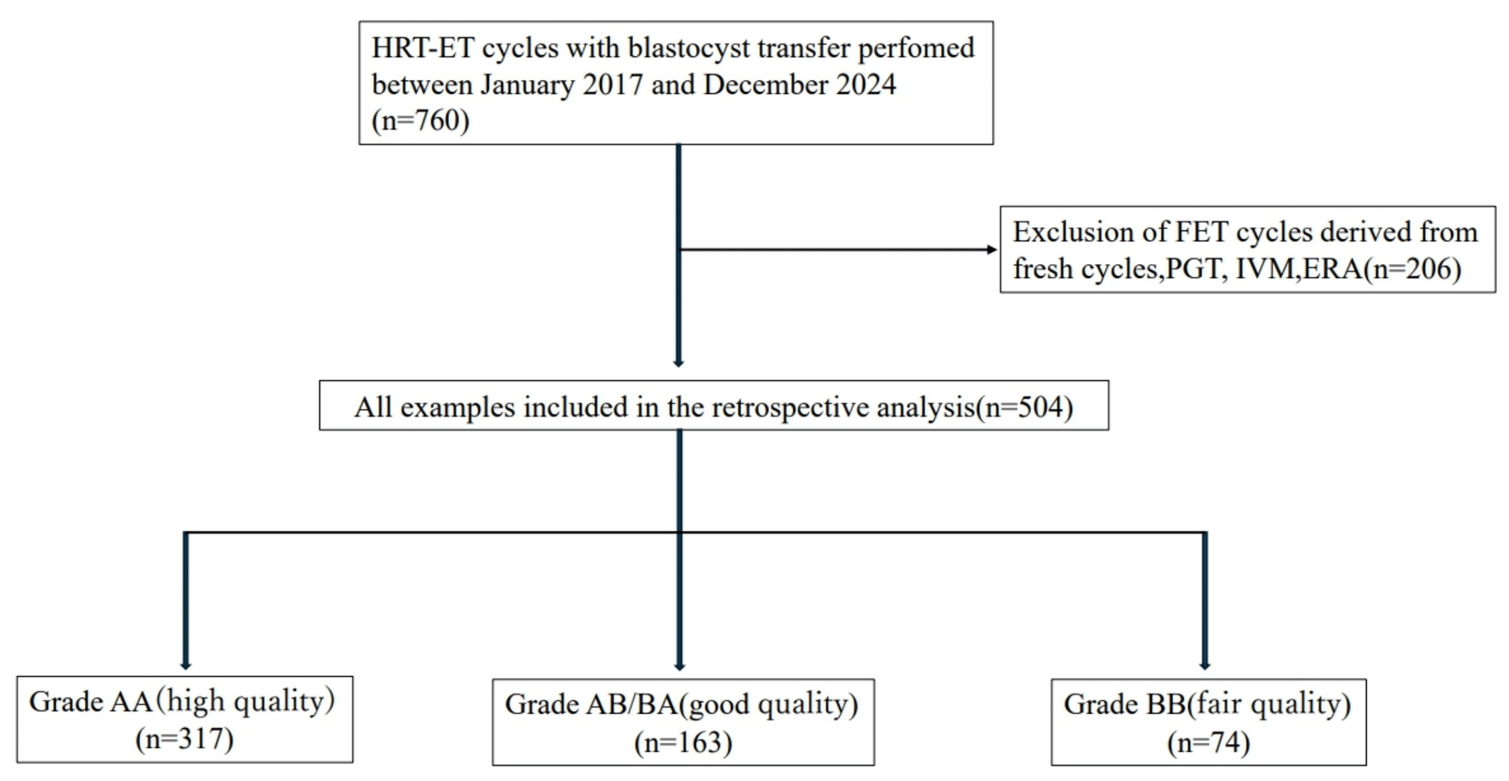
Flow chart showing the inclusion and exclusion criteria for this study Using the Gardner classification to evaluate the quality of blastocysts, grade AA was classified as the best quality, AB/BA group as good quality, and BB group as fair quality. We classified the 554 cases into 317 AA, 163 AB/BA, and 74 BB cases using the Gardner classification. HRT: hormone replacement therapy cycle, FET: frozen embryo transfer, IVM: in vitro maturation, PGT: preimplantation genetic testing, ERA: endometrial receptivity analysis.

We performed a retrospective cohort study of patients who underwent frozen-thawed blastocyst transfers at Fujita Health University Hospital between January 2017 and December 2024. Patient ages ranged from 18-43 years (at the time of embryo freezing). Per our standard protocol, vitrified-warmed blastocyst transfer was performed, with dydrogesterone (Duphaston®) administered orally in our center. The exclusion criteria were as follows: fresh embryo transfer, early embryo transfer, in vitro matured blastocysts using endometrial receptivity analysis (ERA), preimplantation genetic testing (PGT) cycles, and missing data regarding body mass index (BMI) and endometrial thickness on the day of frozen-thawed blastocyst transfer. In total, 554 cases were included, excluding 206 of 760 cases from 298 patients. First, all embryos were analyzed, and no embryo classification was performed. Subsequently, for the subgroup analysis, the embryos were divided into three groups. Using the Gardner classification to evaluate the quality of blastocysts, grade AA was classified as the best quality, AB/BA group as good quality, and BB group as fair quality. We classified the 554 cases into 317 AA, 163 AB/BA, and 74 BB cases using the Gardner classification (Figure 1).

This study was approved by the Institutional Review Board of Fujita Health University, Japan. All patients involved in this study consented to the use of their medical records for research in an unidentifiable manner.

Ovarian stimulation was performed using recombinant or urinary follicle-stimulating hormones. Once the follicles reached 18 mm in size, human chorionic gonadotropin was administered. Oocyte retrieval was performed 36 hours later. All blastocysts were cryopreserved on days five or six. Frozen-thawed embryo transfer was performed under hormone replacement therapy. Transdermal estrogen was administered for 21 days from day two or three following the onset of menses to the day of the pregnancy test. Once serum estradiol concentrations reached ≥ 300 pg/mL and ultrasonic endometrial stripe thickness reached ≥ 8 mm with a proliferative pattern, progesterone was administered, usually via oral dydrogesterone tablets (Duphaston®). Dydrogesterone was administered orally (15 mg three times daily).

Based on the duration of progestin administration, patients were divided into four groups: 120 hours (120 h), 132 hours (132 h), 144 hours (144 h), and 156 hours (156 h). The duration of progestin administration varied depending on the time of year.

We used the Shapiro-Wilk method and the Steel-Dwass test to determine whether there were differences in patients' background age and BMI among the four groups (120 h, 132 h, 144 h, and 156 h). We used Fisher's exact test and the Bonferroni method to determine whether there were differences in the final outcome of pregnancy rate between the four groups (120 h, 132 h, 144 h, and 156 h).

## Results

In the analysis of all embryos, there were no differences in patient background, including age or BMI, at each time point, as determined by the Shapiro-Wilk and Steel-Dwass tests (Table 1).

**Table 1:** Baseline characteristics of the women who underwent frozen-thawed blastocyst transfer at Fujita Health University Hospital between January 2017 and December 2024 in the analysis of all embryos (n=504) The Shapiro-Wilk and Steel-Dwass tests revealed no differences in the patients' background characteristics including age or BMI at any time point. SD: standard deviation, BMI: body mass index

Time (hour)		Age, years (mean ± SD)		BMI, kg/m2 (mean ± SD)	
120		36.9 ± 4.4		20.8 ± 3.5	
132		36.3 ± 4.9		21.8 ± 4.3	
144		36.4 ± 4.9		21.6 ± 4.2	
156		37.4 ± 4.2		21.2 ± 3.8	

In the AA group, there were no differences in age at each time point, as determined by the Shapiro-Wilk and Steel-Dwass tests (Table 2). The 132-h group had a higher BMI than the 120-h group (P<0.0329) (Table 2).

**Table 2:** Baseline characteristics of the women who underwent frozen-thawed blastocyst transfer at Fujita Health University Hospital between January 2017 and December 2024 in the analysis of embryos in the AA group (n=317) The Shapiro-Wilk and Steel-Dwass tests revealed no difference in the patient age at each timepoint. The 132-h group had a higher BMI than the 120-h group (p<0.0329). SD: standard deviation, BMI: body mass index

Time (hour)		Age, years (mean ± SD)		BMI, kg/m2 (mean ± SD)	
120		36.4 ± 4.9		20.1 ± 3.6	
132		35.9 ± 4.7		21.6 ± 3.9	
144		35.4 ± 5.1		21.8 ± 4.5	
156		36.8 ± 4.5		21.1 ± 3.5	

In the AB/BA group, there were no differences in age or BMI at each time point, as determined by the Shapiro-Wilk and Steel-Dwass tests (Table 3).

**Table 3:** Baseline characteristics of the women who underwent frozen-thawed blastocyst transfer at Fujita Health University Hospital between January 2017 and December 2024 in the analysis of embryos in the AB/BA group (n=163) The Shapiro-Wilk and Steel-Dwass tests revealed no difference in the patients' background characteristics including age or BMI at each timepoint. SD: standard deviation, BMI: body mass index

Time (hour)		Age, years (mean ± SD)		BMI, kg/m2 (mean ± SD)	
120		37.8 ± 3.6		22.0 ± 3.1	
132		35.9 ± 5.0		21.5 ± 4.4	
144		37.6 ± 4.3		20.9 ± 3.2	
156		37.8 ± 3.5		20.7 ± 1.8	

In the BB group, there were no differences in age and BMI at each time point, as determined by the Shapiro-Wilk and Steel-Dwass tests (Table 4).

**Table 4:** Baseline characteristics of the women who underwent frozen-thawed blastocyst transfer at Fujita Health University Hospital between January 2017 and December 2024 in the analysis of embryos in the BB group (n=74) The Shapiro-Wilk and Steel-Dwass tests revealed no differences in the patients' background characteristics, including age and BMI at each timepoint. SD: standard deviation, BMI: body mass index

Time (hour)		Age, years (mean ± SD)		BMI, kg/m2 (mean ± SD)	
120		(-)		(-)	
132		37.9 ± 5.4		23.1 ± 5.0	
144		36.4 ± 5.4		23.1 ± 5.2	
156		39.3± 3.4		21.9 ± 5.5	

**Figure 2:**
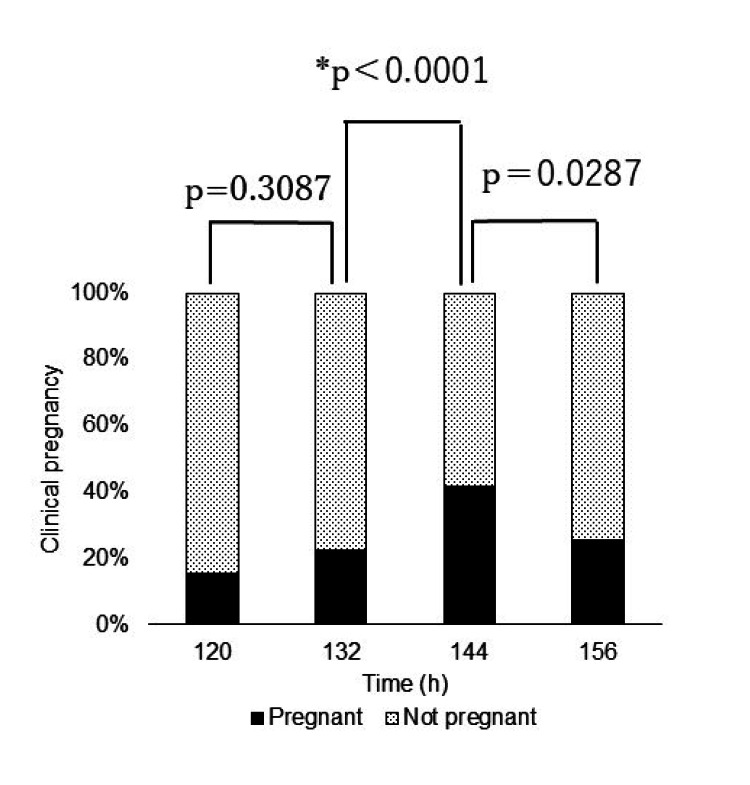
Pregnancy rate in the analysis of all embryos(n=504) First, to determine whether there was a difference in the pregnancy rate at all timepoints, we performed Fisher's exact test and found a significant difference (p=0.0001) at each timepoint. Second, using the Bonferroni method, the pregnancy rate at 144-h group was significantly higher than that at 132-h group (p<0.0001). * Significant difference.

In the analysis of all embryos using Fisher's exact test and the Bonferroni method, the pregnancy rate at each time point of the primary evaluation was significantly higher in the 144-h group than in the 132-h group (p<0.0001) (Figure 2).

**Figure 3:**
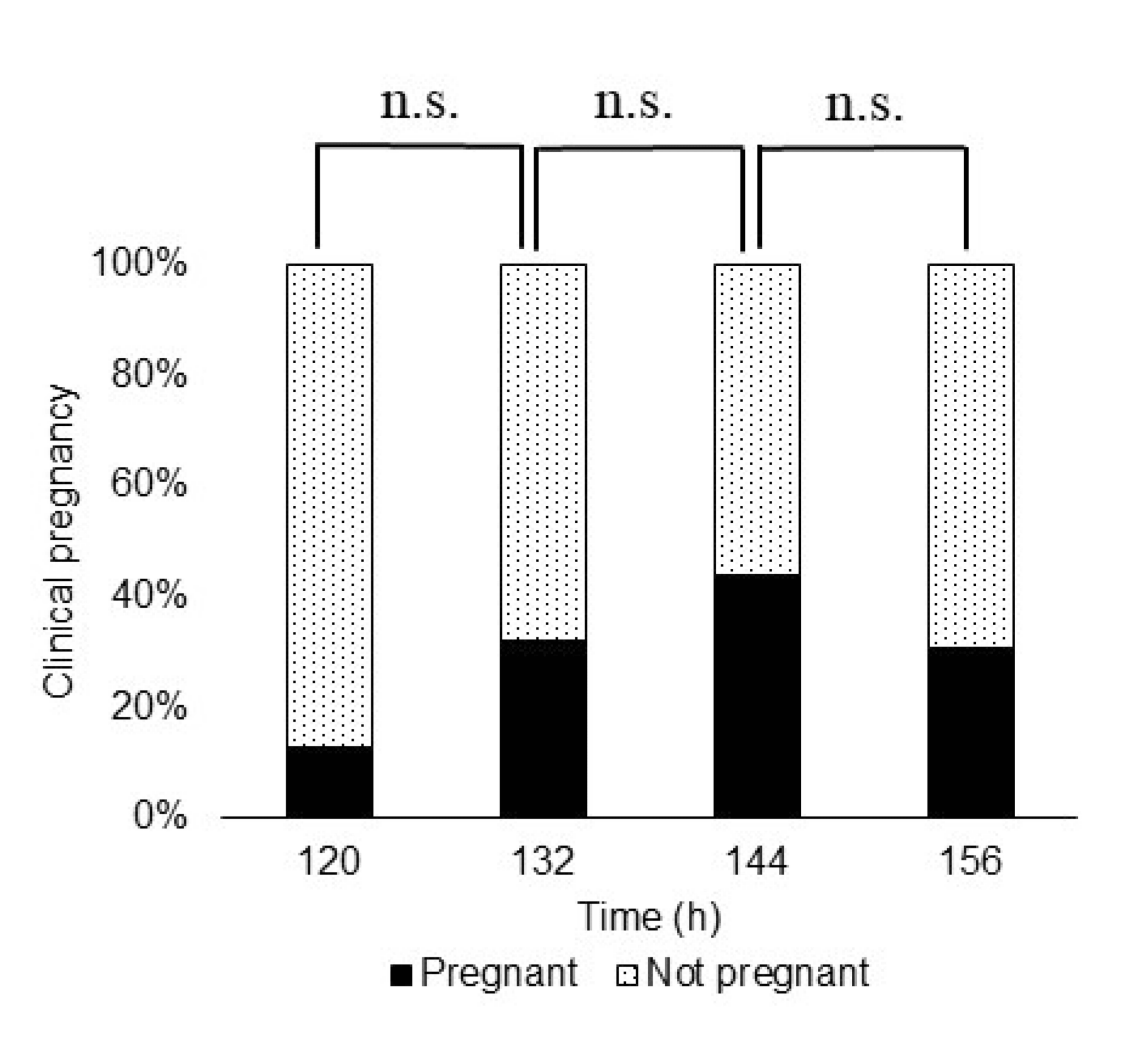
Pregnancy rate in the analysis of embryos in the AA group (n=317) On performing Fisher's exact test to determine whether there was a difference in the pregnancy rate at all timepoints, the p-value for the AA group was 0.059, and no significant difference was found. n.s. = not significant

Next, on analyzing the results by embryo grade in the AA group, Fisher's exact test showed that there was no difference in the pregnancy rate at each time point (Figure 3).

**Figure 4:**
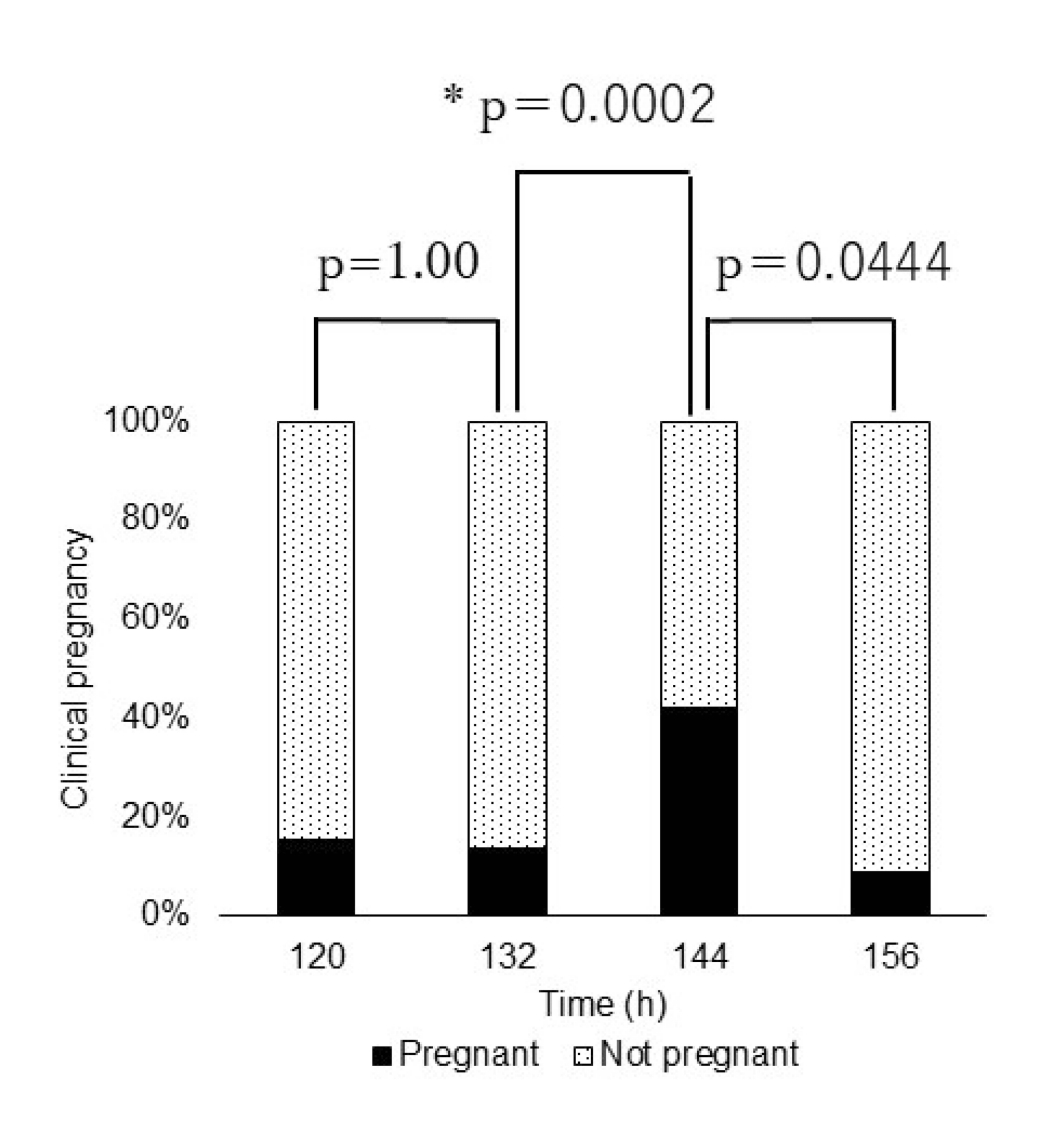
Pregnancy rate in the analysis of embryos in the AB/BA group (n=163) First, we performed Fisher’s exact test to determine whether there was a difference in the pregnancy rate at all time points and found a significant difference (p<0.001) at each timepoint in the AB/BA group. Second, on using the Bonferroni method, we found that the pregnancy rate at 144-h group was significantly higher than that at 132-h group (p=0.0002). * Significant difference.

In the AB/BA group, Fisher's exact test and the Bonferroni method revealed that the pregnancy rate was higher in the 144-h group than in the 132-h group (p = 0.0002) (Figure 4).

**Figure 5:**
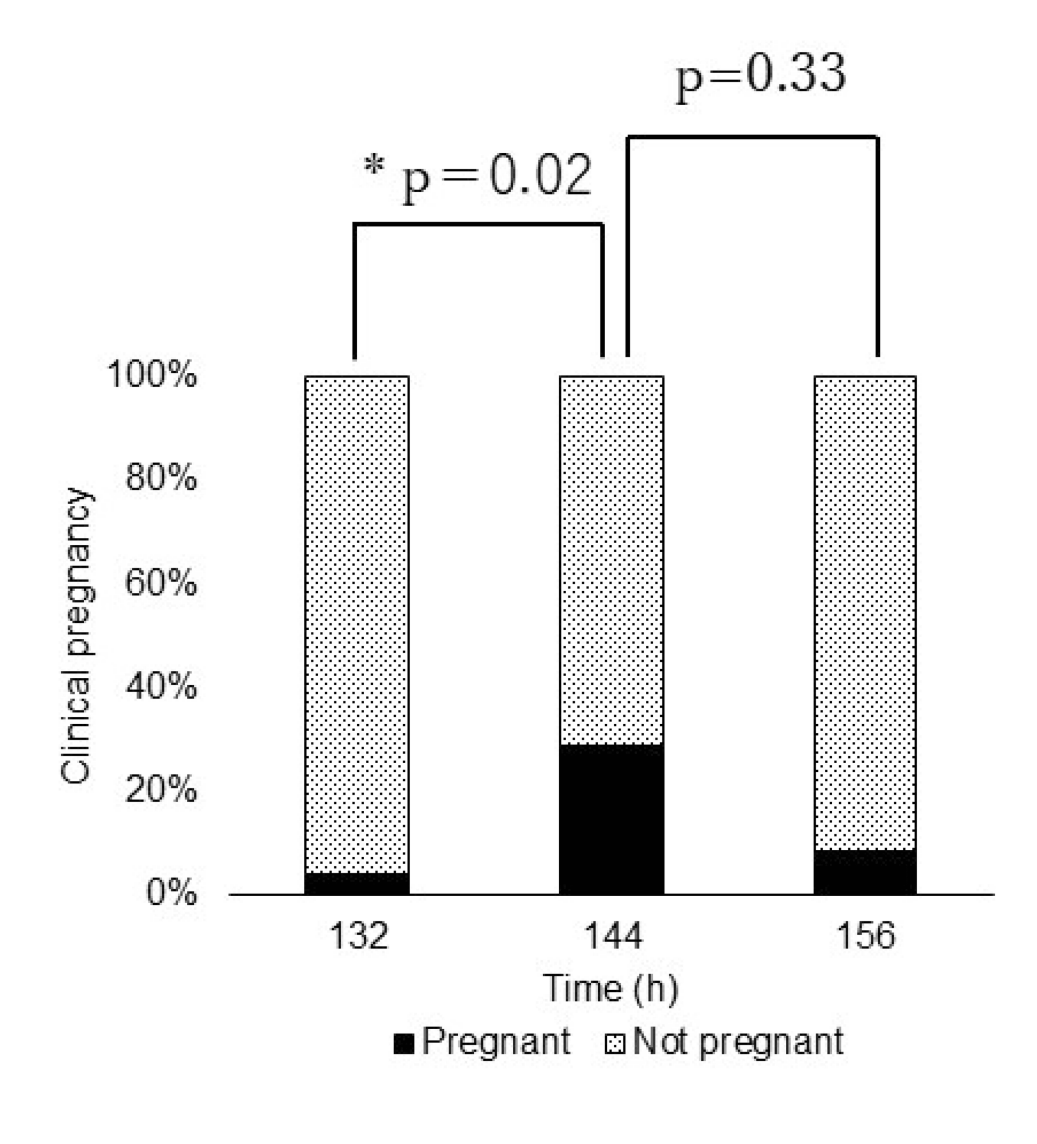
Pregnancy rate in the analysis of embryos in the BB group (n=74) First, we performed Fisher's exact test to determine whether there was a difference in the pregnancy rate at all timepoints, and the result was significant at p=0.0249 in the BB group. Second, on using the Bonferroni method, the pregnancy rate at 144-h group was found to be significantly higher than that at 132-h group (p=0.02).

In the BB group, Fisher's exact test and the Bonferroni method revealed that the pregnancy rate was higher in the 144-h group than in the 132-h group (p=0.02) (Figure 5).

## Discussion

The results of this study clarify two aspects. First, the pregnancy rates in the 144-h group were significantly higher than those in the 132-h group in the analysis of all embryos. Second, WOI may be more important for poor-quality embryos.

This study showed that the oral administration of dydrogesterone requires a WOI of at least 144 hours. The endometrium undergoes dynamic morphological and functional changes in response to estrogen and progesterone that affect implantation. The concept of the WOI was proposed in the 1950s and has since been supported by various studies in the fields of clinical medicine, epidemiology, and morphology [10-13]. The period during which the human endometrium is receptive to implantation is defined as the WOI and is thought to approximately comprise days 19-22 of the mid-secretory phase of the menstrual cycle. The duration of progestin use during a hormone replacement cycle is often 5-7 days. WOI is induced by the presence of exogenous and/or endogenous progesterone following adequate estradiol stimulation. The window is within 30-36 hours during the hormone replacement cycle and varies from patient to patient [1]. It has been reported that WOI is delayed in more than 30% of patients who have undergone embryo transfer [14-16]. There are reports that no differences were observed in the clinical pregnancy rate between 5 and 7 days when vaginal micronized progesterone tablets were administered for warmed blastocyst transfer [17]. However, it has been reported that warmed blastocyst transfer on the sixth and seventh day of progesterone administration during a hormone replacement cycle results in a similar live birth rate. Subgroup analysis revealed a significantly higher miscarriage rate for day six blastocysts transferred on the sixth day of progesterone supplementation than those transferred on the seventh day of progesterone supplementation [18].

In terms of the administration route, the question arises whether oral dydrogesterone is equivalent in efficacy to vaginal micronized progesterone for luteal phase support. One study stated that there was no difference in pregnancy rates between vaginal and oral progesterone preparations [19]. Since vaginal administration can cause implantation failure due to bacterial infection of the uterus, we used oral progesterone instead of vaginal administration until embryo transfer. This study found that the highest pregnancy rate was achieved when progestin was administered for 144 hours or more and that administration for 132 hours was insufficient to achieve pregnancy.

We investigated whether there were differences in pregnancy rates according to grade. There was no significant difference in the pregnancy rate at the four-time points during the luteal hormone administration period in the AA group. However, there was a difference in the pregnancy rates at the four-time points during the luteal hormone administration period in the AB/BA and BB groups. This indicates that the luteal hormone administration period is important for transferring poor-quality embryos. In this study, the pregnancy rate was higher when progesterone was administered for 144 hours or more, but in all studies, there was no difference in the pregnancy rate between 144-h group and 156-h group; therefore, it is not the case that the longer the administration time, the better the results. ERA is a test used to determine the optimal WOI for each embryo. When ERA is performed in patients with recurrent implantation failure, it is believed that approximately 70% of women are receptive, and the remaining 30% are nonreceptive. For nonreceptive women, the implantation rate can be improved by modifying the timing of the transfer [20]. Simon et al. conducted a prospective study in which embryos were divided into three groups: fresh embryo transfer, blastocyst transfer, and blastocyst transfer after ERA. The cumulative pregnancy rate for blastocyst transfer after ERA was significantly higher than that for the other methods [21]. In 2018, Tan et al. reported individualized embryo transfer of euploid embryos after ERA. Among the patients who did not become pregnant after ERA, 62.5% were receptive and 37.5% were non-receptive. The pre-receptive and post-receptive cases accounted for 83.3% and 5.6% of the non-receptive cases, respectively. Next, when single embryo transfer was performed in 17 women who had undergone ERA, there was no significant difference in the implantation rate or pregnancy continuation rate between the ERA and non-ERA groups; however, this was a retrospective study with a small number of cases, and therefore, it would be desirable to increase the number of cases in future studies [22]. Recently, an ER Map, a molecular tool for assessing human endometrial receptivity, was developed based on the transcriptome analysis of genes related to endometrial proliferation and implantation. Accordingly, it was reported that most patients became receptive by the fifth or sixth day of progesterone administration, but some patients became receptive as early as on the 2.5th day of progesterone administration, and others became receptive on the eighth day of progesterone administration [23]. However, because gene transcriptome analysis is expensive and invasive, it is not considered beneficial in all cases. Therefore, the administration period before embryo transfer should be at least 144 hours during hormone replacement cycles when using dydrogesterone for the first embryo transfer.

This study has several limitations that should be considered when interpreting these results. The retrospective, single-center design limits the assessment of causality and introduces selection bias, which limits generalizability. As there is a report that says "When endometrial thickness was ≥ 8 mm, patients were injected intramuscularly with progesterone (80 mg/day) for 3 to 7 days, three days of progesterone regimen prior to day three embryo transfer may be more beneficial", the optimal duration of progesterone administration may differ depending on the type of progesterone hormone and the route of administration [24]. In this study, dydrogesterone was administered orally, but the optimal duration of administration may differ depending on the administration route and type of progesterone; therefore, further research is needed.

## Conclusions

The results of this study clarify two aspects. First, the pregnancy rate in the 144-h group was significantly higher than that in the 132-h group in the analysis of all embryos. Second, WOI may be more important for poor-quality embryos. This study showed that the oral administration of dydrogesterone requires a WOI of at least 144 hours.
